# DSGRN: Examining the Dynamics of Families of Logical Models

**DOI:** 10.3389/fphys.2018.00549

**Published:** 2018-05-23

**Authors:** Bree Cummins, Tomas Gedeon, Shaun Harker, Konstantin Mischaikow

**Affiliations:** ^1^Department of Mathematical Sciences, Montana State University, Bozeman, MT, United States; ^2^Department of Mathematics, Rutgers, The State University of New Jersey, New Brunswick, NJ, United States

**Keywords:** Boolean networks, switching systems, network dynamics, parameter space, database of dynamics

## Abstract

We present a computational tool DSGRN for exploring the dynamics of a network by computing summaries of the dynamics of switching models compatible with the network across all parameters. The network can arise directly from a biological problem, or indirectly as the interaction graph of a Boolean model. This tool computes a finite decomposition of parameter space such that for each region, the state transition graph that describes the coarse dynamical behavior of a network is the same. Each of these parameter regions corresponds to a different logical description of the network dynamics. The comparison of dynamics across parameters with experimental data allows the rejection of parameter regimes or entire networks as viable models for representing the underlying regulatory mechanisms. This in turn allows a search through the space of perturbations of a given network for networks that robustly fit the data. These are the first steps toward discovering a network that optimally matches the observed dynamics by searching through the space of networks.

## 1. Introduction

Experimentally determined pairwise interactions between genes, proteins and signaling molecules are often assembled into pathways and networks. There is a strong desire to understand the dynamics of networks, diversity of their potential stable behavior, as well their response under mutations or targeted pharmacological intervention. Such an ability would allow us to target many diseases, most importantly cancer, with great precision and accuracy, without disturbing other functions of the cell, and without the devastating side effects on healthy cells that are the hallmark of many current drugs.

The current state of modeling gene network dynamics is characterized by a trade-off between the model's ability to quantitatively match the experimental data, and the need for a large number of kinetic parameters to parameterize the model (Karlebach and Shamir, 2008; Heatha and Kavria, 2009; Machado et al., 2011; Goncalves et al., 2013). Properly parameterized ordinary differential equation models can provide a good quantitative match and are easily generalized (Chen et al., 2004; Tyson and Novak, 2013). However, numerical simulation of these models require knowledge of kinetic parameters that are usually not known. The indirect estimate of these parameters by comparing the output of the model to the experimental data suffers from at least three fundamental problems: (i) the correspondence between dynamics and the structure of the network is not one-to-one; (ii) the need to match data corrupted by significant intrinsic and experimental noise to an individual solution of the ODE model; and (iii) the lack of methods to search high dimensional parameter spaces for dynamic signatures observed in the data.

A popular modeling platform is that of Boolean nets, where each protein, ligand or mRNA is assumed to have two states (ON and OFF), and the discrete time evolution of the states is based on logic-like update functions (Glass and Kauffman, 1972, 1973; Thomas, 1973; Thomas et al., 1995; von Dassow et al., 2000; Bernard and Gouze, 2002; de Jong, 2002; de Jong et al., 2004; Belta and Habets, 2006; Chaves et al., 2006; Faure et al., 2006; Albert, 2007; Batt et al., 2007a,b; Bornholt, 2008; Tournier and Chaves, 2009; Machado et al., 2011; Albert et al., 2013; Saadatpour and Reka, 2013). Rather than providing rate parameters, the biological input into model formulation is limited to postulating logical functions, one for each node in the network, which compute the next Boolean state of node *i* based on Boolean states of the nodes that provide input to node *i*. These Boolean functions at each node are assembled into a Boolean function that provides the next state of all nodes in the network based on the previous state of the network. Iterations of this function are an approximation of the time evolution of the state of the network.

This attractive class of synchronous Boolean models has several disadvantages. The first class of objections comes from biology: these models cannot represent ubiquitous cellular noise, since states change simultaneously they require unreasonable uniformity of duration of different cellular processes, and the fit to experimental data is typically problematic. A mathematical objection is that discretization of the phase space and the discretization of the set of Boolean functions compatible with a given network does not allow consideration of changing dynamics under graded perturbation. In other words, it is difficult to construct a bifurcation theory in the class of Boolean functions.

In this contribution we study multi-level discrete maps, which are a direct generalization of Boolean maps, that are compatible with an ODE system. We propose that only the asynchronous updates of these discrete maps have biological meaning. The concept of an asynchronous update of a Boolean function has been introduced previously (Pauleve and Richard, 2012). We review and formalize these concepts in the next section. We then study a particular class of ODEs that can be viewed as a continuous parameterization of a family of multi-level discrete maps. Continuous parameterization of a finite number of inherently discrete objects implies that there is a finite decomposition of the parameter space into disjoint domains, each of which supports a multi-level discrete map. Mutual position of these parameter domains is captured in a *parameter graph*, whose nodes represent the domains and edges their adjacency.

We describe a computational approach, called Dynamic Signatures Generated by Regulatory Networks (DSGRN), that computes the parameter graph for a given network and input interaction at each node. In addition, to each node of the parameter graph we associate a *Morse graph* whose nodes are the strongly connected path components of the asynchronous update of the corresponding multi-level discrete map, and edges represent reachability by iterations of this map. We call the resulting collection a *DSGRN Database*.

## 2. Basic definitions

**Definition 2.1.** A *regulatory network* **RN** = (*V, E*) is a graph with network nodes *V* = {1, 2, …, *N*} and signed, directed edges *E* ⊂ *V* × *V* × {→, ⊣}. For *i, j* ∈ *V*, we will use the notation (*i, j*) ∈ *E* to denote a directed edge from *i* to *j* of either sign, *i* → *j* to denote an *activation* or positive interaction, and *i* ⊣ *j* to denote a *repression* or negative interaction.

We define the *targets* of a node *i* as

T(i):  ={j∣(i,j)∈E}

and the *sources* of a node *i* as

S(i):  ={j∣(j,i)∈E}

For each node *i* in a regulatory network **RN**, define a set of *integer states* $V\left(i\right):=\left\{0,1,\ldots ,m_{i}\right\}$. Let $V:=\prod_{i=1}^{N}V\left(i\right)$. For state s∈V let

S+i:  ={u∈V | ui>si,uj=sj for all j=i}

be the set of states that differ from *s* only in the *i*-th coordinate and are strictly greater in the *i*-th coordinate.

**Definition 2.2.** We say a (multi-valued) map f:V→V is *compatible* with a regulatory network **RN** (**RN**-compatible) if and only if the following are satisfied

- (*i, j*) ∈ *E* is a positive edge *i* → *j* if and only if there exists a state s∈V and at least one $u\in S_{+}^{i}$ such that *f*_*j*_(*u*)>*f*_*j*_(*s*).
- (*i, j*) ∈ *E* is a negative edge *i*⊣*j* if and only if there exists a state s∈V and at least one $u\in S_{+}^{i}$ such that *f*_*j*_(*u*) < *f*_*j*_(*s*).

A regulatory network, as introduced in this paper, is also called the *interaction graph* of Boolean function *f*, as defined in Pauleve and Richard (2012). Our definition above goes in the opposite direction and defines a set of multivalued maps consistent with a fixed regulatory network; we also generalize from Boolean maps to maps with more than two discrete values.

**Definition 2.3.** A *synchronous Boolean model for a regulatory network* ***RN*** is an **RN**-compatible map

$$B:{\left\{0,1\right\}}^{N}\to {\left\{0,1\right\}}^{N}.$$

Given a synchronous Boolean model *B*, the regulatory network **RN** such that *B* is **RN**-compatible, is the *interaction graph of* *B*.

**Definition 2.4.** A *synchronous multi-level discrete map for a regulatory network* ***RN*** is an **RN**-compatible map

D:V→V

where $V=\prod_{i=1}^{N}\left\{0,1,\ldots ,m_{i}\right\}$.

**Definition 2.5.** A *nearest neighbor multi-valued map for a regulatory network* ***RN*** is an **RN**-compatible map

F:V⇉V

such that either s∈F(s) or, if v∈F(s) and *v* ≠ *s* then *v* satisfies the *adjacency condition*:

$$|v_{i}-s_{i}|=\{\begin{array}{cc} 1, & \text{for }i=k\\ 0, & \text{for }i\neq k \end{array}$$

for exactly one index *k*. We say that *s* and *v* are *adjacent*.

**Definition 2.6.** We say a nearest neighbor multi-valued map F is *an asynchronous update* of a multi-level discrete map *D* if, given

$$t_{1}=D(s_{1})\text{ where }t_{1}=(t_{1,1},\ldots ,t_{1,N})\text{ and }s_{1}=(s_{1,1},\ldots ,s_{1,N}),$$

we have $s_{2}\in F\left(s_{1}\right)$ in either of the two following conditions:

- if *t*_1_ = *s*_1_ then *s*_2_ = *s*_1_; or
- if *t*_1_ ≠ *s*_1_, then *s*_2_ is adjacent to *s*_1_, and *s*_2_ lies between *s*_1_ and *t*_1_, which means that either
  - *s*_1,*i*_ < *s*_2,*i*_ ≤ *t*_1,*i*_ or
  - *s*_1,*i*_ > *s*_2,*i*_ ≥ *t*_1,*i*_.

For a regulatory network **RN** = (*V, E*) consider a system of ODEs in variables *x*_*i*_ for each *i* ∈ *V*. We assume that there are finite number of thresholds θ_1,*i*_, …, θ_*m*_*i*_, *i*_ that divide the semi-axis [0, ∞) to *m*_*i*_ + 1 intervals *I*_*k*_. The collection of thresholds {θ_*j, i*_} decomposes [0, ∞)^*N*^ into a finite number of domains κ, characterized by the property that the projection on *i*-th variable π_*i*_(κ) = *I*_*k*_ for a unique *k* ∈ {0, …, *m*_*i*_} for every *i*. We call each κ a *domain*. Let K be a collection of all domains κ ⊂ ℝ^*N*+^ in the non-negative orthant of ℝ^*N*^.

Let $x=\left(x_{1},\ldots ,x_{N}\right)\in {\mathbb{R}}^{N+}$ and let

$$G_{i}:[0,\infty )\to V(i)$$

be defined by *G*_*i*_(*x*_*i*_) = *k* if and only if *x*_*i*_ ∈ *I*_*k*_. Let

$$G:{\left[0,\infty \right)}^{N}\to V$$

be the vector-valued function with coordinate functions *G*_*i*_. For a given domain κ, the value *G*(*x*) does not depend on *x* ∈ κ. Therefore we can assign the *state* s:=G(x)∈V,x∈κ to the domain κ and write *s* = *g*(κ). Viewed as a map on the set of domains K, *g* is a bijection

g:K→V.

**Definition 2.7.** For a regulatory network **RN** = (*V, E*) consider a system of ODEs in variables *x*_*i*_ for each *i* ∈ *V*. We say that such an ODE system is *compatible* with a nearest neighbor multi-valued map F if solutions *x*(*t*) can traverse from domain κ_1_ to adjacent domain κ_2_ only if $g\left(\kappa_{2}\right)\in F○g\left(\kappa_{1}\right)$.

This definition of compatible ODE system states that the dynamics of an ODE system can be captured, in an coarse sense, by a finite multi-valued map. We now apply these ideas to a specific family of ODE systems.

## 3. Switching systems

Switching systems, also known as Glass systems, were introduced by Glass (Glass and Kauffman, 1972, 1973) in the 1970's and developed subsequently by many authors (Thomas, 1973; Thomas et al., 1995; Edwards, 2001; Bernard and Gouze, 2002; de Jong, 2002; de Jong et al., 2004; Chaves et al., 2006; Tournier and Chaves, 2009; Ironi et al., 2011; Edwards et al., 2015).

**Definition 3.1.** A *switching system* for a regulatory network **RN** = (*V, E*) is a system of ordinary differential equations

(1) $${\dot{x}}_{i}=-\gamma_{i}x_{i}+M_{i}○\sigma_{i}(x),i\in V$$

where γ_*i*_ > 0 is a decay rate, *M*_*i*_ is a multi-affine algebraic expression (Belta and Habets, 2006; Batt et al., 2007b; Cummins et al., 2016), and σ_*i*_ = (σ_*i, j*_) is a vector of step functions, one for each edge (*j, i*) ∈ *E*. When (*j, i*) = *j* → *i* is an activation, then the step function transitions from a low (*l*_*i, j*_) to a high value (*u*_*i, j*_), and when (*j, i*) = *j* ⊣ *i* is a repression, then σ_*i, j*_ transitions from *u*_*i, j*_ to *l*_*i, j*_. The transition happens at the threshold *x*_*j*_ = θ_*j, i*_:

(2) $$\sigma_{i,j}:\;=\{\begin{array}{cc} l_{i,j} & \text{if }j\to i\in E\text{ and }x_{j}<\theta_{i,j}\\ & \text{or }j⊣i\in E\text{ and }x_{j}>\theta_{i,j}\\ u_{i,j} & \text{if }j\to i\in E\text{ and }x_{j}>\theta_{i,j}\\ & \text{or }j⊣i\in E\text{ and }x_{j}<\theta_{i,j} \end{array}$$

We assume 0 < θ_*i, j*_ and 0 < *l*_*i, j*_ < *u*_*i, j*_ to ensure the model captures the basic biological meaning of concentration, activation, and repression. We further assume θ_*i,j*_ ≠ θ_*k, j*_ for all *j* ∈ *V* whenever *i* ≠ *k* and so each node *j* affects its downstream nodes at different thresholds.

It is important to note that to a given **RN** one can associate many switching systems. Indeed, a selection of multi-linear expressions *M*_*i*_, *i* = 1, …, *N* in addition to the structure of the network **RN**, determines the parameterized set of ODEs (1). The function *M*_*i*_ determines how the information from the source nodes **S**(*i*) is combined into the right hand side of (1).

A *parameter* of the switching system is a set of real numbers

$$p=\{\gamma_{i}\;|\;i\in V\}\cup \{\theta_{i,j},l_{i,j},u_{i,j}\;|\;(j,i)\in E\}$$

that satisfy these constraints. The set of all parameters *p* is denoted *P*.

**Definition 3.2.** The collection Θ_*i*_: = {θ_*j, i*_ | *j* ∈ **T**(*i*)} for each node *i* ∈ *V* is totally ordered, and this order induces a decomposition of phase space K, such that each domain κ∈K is written

$$\kappa =\prod_{i}\left[\theta_{jk,i},\theta_{j_{k+1,}i}\right]$$

where θ_*j*_*k*_,*i*_, θ_*j*_*k*+1_,*i*_ are adjacent. We define the thresholds θ_0,*i*_: = 0 and θ_∞,*i*_: = ∞, so that the intervals below the lowest threshold and above the highest threshold are captured.

Let *m*_*i*_ = |**T**(*i*)| be the number of targets of node *i* ∈ *V*, and let $V=\prod_{i=1}^{N}\left\{0,1,\ldots ,m_{i}\right\}$ as before. The decomposition K is the same as that in the previous section, and using the total order on Θ_*i*_, we can construct an appropriate bijection g:K→V. Using this bijection *g*, we show in Crawford-Kahrl et al. (2018) that given a switching system at a fixed parameter *p* ∈ *P*, there is a unique multi-level discrete map *D*^*p*^, and an asynchronous update rule of *D*^*p*^, $F^{p}$, such that the switching system is compatible with $F^{p}$. We note that the collection ${\left\{D^{p}\right\}}_{p\in P}$ does not exhaust the entire collection of **RN**-compatible multi-level maps *D*. However, the induced collection of maps ${\left\{D^{p}\right\}}_{p\in P}$ decomposes into finite number of classes.

**Definition 3.3.** Let *p* be a parameter of a switching system with totally ordered thresholds $\Theta_{i}^{p}$. Let *D*^*p*^ be the unique multi-level function associated to the switching system parameterized by *p*. Let $O_{i}^{p}=\left\{j_{1}<j_{2}<\cdots <j_{m_{i}}\right\}$ be such that *j*_*k*_ < *j*_*l*_ if and only if θ_*j*_*k*_, *i*_ < θ_*j*_*l*_, *i*_ in $\Theta_{i}^{p}$. Define $O^{p}=\left\{O_{i}^{p}\right\}$ to be the *order parameter* associated to *p*, and (*O*^*p*^, *D*^*p*^) to be the *combinatorial parameter* of the system. If *q* is another parameter of the switching system with (*O*^*q*^, *D*^*q*^), then we define an equivalence relation *q* ~ *p* when (*O*^*q*^, *D*^*q*^) = (*O*^*p*^, *D*^*p*^). We call the collection of combinatorial parameters Z.

The partition induced by ~ is clearly finite, since the order of *m*_*i*_ integers is finite, and the number of multi-level maps *D* on a finite set is also finite. Let s:=|Z| be the cardinality of the set Z. We show in Cummins et al. (2016) that each u∈Z has a computable geometrical representation as a connected subset *U* ⊂ *P*. Therefore there is a computable decomposition of the parameter space *P* in *s* regions *U*_*i*_ for *i* = 1, …, *s*, such that for any *p, q* ∈ *U*_*i*_ we have *D*^*p*^ = *D*^*q*^, and hence also $F^{p}=F^{q}$. Therefore a finite collection ${\left\{F^{u}\right\}}_{u\in Z}$ captures dynamics of all maps $F^{p}$ across all the parameter space *P*.

We remark that the parameter graph captures the dynamics of all subgraphs of **RN** as well as **RN** itself. Although not addressed in this paper, we can limit the exploration of the dynamics only to those combinatorial parameters that result in **RN**-compatible multi-level discrete maps *D*.

## 4. DSGRN: dynamical signatures generated by regulatory networks

Given a network **RN** and the associated switching system, the computational tool DSGRN (Cummins et al., 2016; Harker, 2018) computes and records a graph of graphs in SQL database format. This general database can be queried in many ways, and we will give a short example after defining the graphs that are computed. If a user starts with a synchronous Boolean model *B*, the first step is to calculate an the interaction graph **RN** of *B*. DSGRN then describes the long term dynamics of all multi-valued nearest neighbor maps compatible with the switching systems associated to **RN.** Each of these multi-valued nearest neighbor maps is an asynchronous update of a multi-level discrete map. Therefore DSGRN embeds the dynamics of *B* into a family of multi-level discrete models that are all compatible with the dynamics of a switching system associated to **RN**.

**Definition 4.1.** The parameter graph P=(C,A) has nodes *C* that represent all combinatorial parameters via a bijection h:C→Z. The non-directed edges (*c, c*′) ∈ *A* occur when the difference between *h*(*c*) = (*O, D*) and *h*(*c*′) = (*O*′, *D*′) is exactly one of the following:

1. there is a swap in the order of one pair of adjacent integers *j*_*k*_, *j*_*l*_ between *O* and *O*′, and all other elements remain the same;
2. for exactly one v∈V, ||*D*(*v*)−*D*(*v*′)|| = 1, and ||*D*(*w*)−*D*′(*w*)|| = 0 for all w∈V\{v}.

For each u∈Z, there is a representative nearest-neighbor multi-valued discrete map $F^{u}$. This map can be viewed as a graph.

**Definition 4.2.** The *state transition graph (STG)* of a switching system with combinatorial parameter *u* is the directed graph (V,E), where the nodes V were defined previously, and (v,w)∈E if and only if $w\in F^{u}\left(v\right)$.

A *recurrent component* (also referred to as a *strongly connected path component*) of the STG (V,E) is a maximal collection M of vertices such that for any u,v∈M there exists a non-empty path from *u* to *v* within the subgraph induced by M. The collection of all recurrent components of (V,E) is denoted by

MD(F):  ={M(i)⊂V∣i∈P}

and is called a *Morse decomposition* of the STG. Here P is an index set. Recurrent components inherit a well-defined partial order by the reachability relation in the directed graph (V,E). In particular, there is a partial order on the indexing set P of MD(F) defined by i ≤ j if there exists a path in (V,E) from an element of M(j) to an element of M(i).

**Definition 4.3.** The *Morse graph* of the STG, denoted MG(F), is the Hasse diagram of the poset (P, ≤). We refer to the elements of P as the *Morse nodes* of the graph.

Any recurrent behavior of the ODE system will be be captured by one of the Morse nodes of the Morse graph. That is, any recurrent set of the ODE will be a subset of a set of domains that correspond to states in STG that belong to a single Morse node.

Each component of the Morse graph can be annotated. We use the following terminology:

1. FP denotes a Morse graph component consisting of a single node of the state transition graph (STG).
2. FP(*v*) denotes an FP that is located in κ = *g*^−1^(*v*) for v∈V.
3. FP ON denotes an FP in which the associated *v* has no zeros.
4. FP OFF denotes an FP in which the associated *v* is all zeros.
5. FC denotes a Morse graph component M that contains at least one path through the subgraph induced by M that crosses at least one threshold in each variable *x*_*i*_. *FC* stands for “full cycle.”
6. XC(*x*_*j*_1__, …, *x*_*j*_*n*__) denotes a partial oscillation in variables *x*_*j*_1__, …, *x*_*j*_*n*__, where only thresholds in these variables are crossed by paths in the Morse graph component.

If a component is a leaf of the Morse graph, i.e., it has no outgoing edges, then we call it an *attractor*. For each node in the parameter graph, DSGRN records the annotated Morse graph, and this collection comprises the database.

## 5. Example

**Figure 1:**
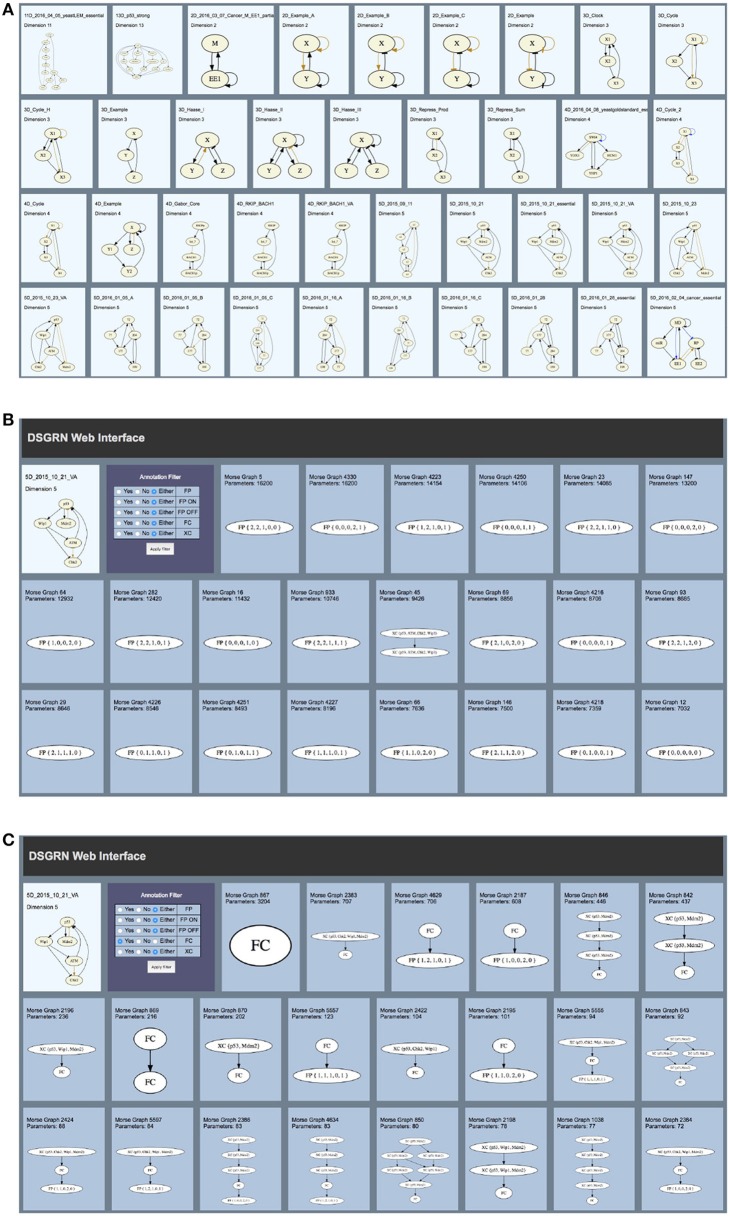
Screenshots of http://chomp.rutgers.edu/Projects/DSGRN/DB/index.html. The description of the Figure and step-by-step guide through an example is in the text.

A DSGRN Database can queried via any general expression in SQL. Some queries have been implemented on a sample set of databases at http://chomp.rutgers.edu/Projects/DSGRN/DB/index.html. See Figure 1A for a screenshot of the above website showing networks with precomputed databases. This screenshot shows a selection of different regulatory networks, each of which may be clicked on to show detailed information about the computation of the network dynamics. Figure 1B shows a screenshot the result of such a click, and Figure 1B shows the result of applying a filter to the network dynamics. We will now step through each of these screenshots in more detail to explain the displayed summary of network dynamics.

In Figure 1A, in the third row on the right, we see a network labeled 5D_2015_10_21_VA. Clicking on it, we see the middle screenshot in Figure 1B. The picture of the network **RN** is in the upper left, and next to it an Annotation Filter, which allows us to filter the results based on the annotations of the displayed Morse graphs. All of the annotated Morse graphs that are generated by at least one combinatorial parameter are shown, ordered by the number of combinatorial parameters that produced the given Morse graph. By clicking on the “Yes” button beside FC, we select the Morse graphs that contain a component annotated by *FC*. In Figure 1C, we show a few top Morse graphs satisfying this condition. By choosing different combinations of “Yes”, “No”, and “Either” in the Annotation Filter, we can explore the different dynamical behaviors of the system.

Although graphical display of the database is useful for exploratory purposes, it is not as powerful as SQL searches over the DSGRN database in which arbitrary combinations of annotated Morse graphs can be selected. Moreover, to use graphical display it is necessary to set up a server. The expected use of DSGRN is to calculate the database and then to use flexible, user-defined SQL queries to search for dynamics of interest.

We now show how to perform some queries that are not available in our demo website. In order to compute the database for DSGRN, the user needs to install DSGRN (Harker, 2018) from GitHub, following the instructions on http://dsgrn.readthedocs.io/en/latest/index.html. While we intend to provide SBML compatibility in the near future, currently the user needs to create a network file that provides names for each node in the regulatory network **RN** and describes the input logic function *M*_*i*_ for each node *i*. The following is the network file for 5D_2015_10_21_VA as shown in the upper left of the middle screenshot in Figure 1B:

p53 : (Chk2 + ATM)(~Mdm2)

ATM : ~Wip1

Chk2 : ATM (~Wip1)

Wip1 : p53

Mdm2 : p53

The name of the node is on the left hand side of the colon, and the input logic function *M*_*i*_ to the node is on the right hand side. For example, p53 has three inputs, with “OR” (addition) logic between Chk2 and ATM, and “AND NOT” (multiplication) logic on Mdm2. The symbol “~” denotes repression. Suppose that this file is saved under “RN.txt.” To compute the DSGRN SQL Database named “RN.db” using 4 threads we run the following command:

mpiexec -np 4 Signatures RN.txt RN.db

After the database is computed, we can query RN.db for different dynamical behaviors. Several tables for the database are automatically generated, including Signatures, MorseGraphAnnotations, and MorseGraphEdges, which we will use in queries below. For a comprehensive list of the tables generated, more detail on the SQL database, and other queries, see the links from the documentation site http://dsgrn.readthedocs.io/en/latest/index.html.

We take the number of combinatorial parameters that generates a specific dynamical behavior to be a proxy for the robustness of the behavior across all of parameter space. The number of combinatorial parameters for network **RN** specified in RN.txt is the number of rows in the database RN.db. Therefore we can find the number of parameters using the command:

sqlite3 RN.db ‘select count(^*^) from Signatures’

which in this case tells us that there are 803,520 parameters associated to the network 5D_2015_10_21_VA. We now search the database for the number of combinatorial parameters with at least one *stable* FC. Note that the Annotation Filter in Figure 1B searches for any FC, including unstable ones. The command for this search is

sqlite3 RN.db ‘select count(^*^) from

Signatures natural join

(select distinct(MorseGraphIndex) from

(select MorseGraphIndex,Vertex from

MorseGraphAnnotations where Label="FC"

except select MorseGraphIndex,Source from

MorseGraphEdges))'

and the result is 6904 combinatorial parameters, which is 0.86% of all the parameters. In contrast, the number with at least one stable FP is 667,536, which is 83% of the parameters, obtained by:

sqlite3 RN.db ‘select count(^*^) from

Signatures natural join

(select distinct(MorseGraphIndex) from

(select MorseGraphIndex,Vertex from

MorseGraphAnnotations where Label like

"FP%"

except select MorseGraphIndex,Source from

MorseGraphEdges))'

Based on the results of these queries, we conclude that a stable FP is far more common that a stable FC, and therefore a more robust behavior for this network.

Table 1 shows the computational scaling of DSGRN in a series of small networks taken from http://chomp.rutgers.edu/Projects/DSGRN/DB/index.html, some of which are shown Figure 1A. We see that the computation time and database storage increase rapidly as the network size increases. This increase is due particularly to the presence of high degree nodes, rather than to the absolute number of nodes and edges. High degree nodes cause the most rapid increase in the number of combinatorial parameters. Because of parallelization and usage of computing clusters with a large core count, we find in practice that DSGRN is more limited by space to store databases than by computation time.

**Table 1:** Example performance of DSGRN on 4 threads on a 2013 MacBook Pro. In practice, DSGRN is limited more by storage space than by computation time.

**Name**	**# Nodes**	**# Edges**	**# Parameters**	**Time**	**Storage**
2D_Example_A	2	4	1,600	2.7 s	124 K
3D_Cycle	3	5	5,400	3.1 s	224 K
4D_Example	4	6	122,472	10.4 s	4 M
5D_2015_10_21_VA	5	8	803,520	2 m 26 s	46 M
7D_2016_04_05_yeastLEM	7	10	3,499,200	12 m 41 s	128 M

In order to address the storage space scaling limitations, we have implemented two additions to DSGRN. The first is the idea of “essential” parameters, which is the subset of parameters consistent with Definition 2.2. DSGRN was originally designed to study not only **RN**-compatible asynchronous multi-level maps, but all such maps that were **S**-compatible with any subgraph **S** of **RN.** By limiting ourselves to **RN**-compatible maps, the size of parameter space is greatly reduced. To specify essential parameters, add “: E” to the end of every line in the network specification file for **RN.** For example, the essential network specification file for 2D_Example_A using multiplicative logic is:

X : XY : E

Y : XY : E

The second addition is an extensive Python module DSGRN that can be used to explore individual parameters rather than calculating the entire database at once. This model is part of the standard DSGRN installation. If a hypothesis about the network dynamics can be constructed a priori, then the selection for annotated Morse graphs can be computed on the fly, allowing much larger networks to be analyzed than is otherwise possible. See https://github.com/shaunharker/DSGRN/blob/master/Tutorials/GettingStarted.ipynb for a brief introduction to the Python library.

## 6. Discussion

Given a regulatory network **RN** there is a very large number of multi-level maps *D* that can be associated to this network. We can enumerate them by selecting for each node an arbitrary assignment of node value based on the node inputs. If the structure of the network is the only information available, these all represent valid models for the network dynamics in the class of discrete multi-level maps, which generalize Boolean models. This class of functions generate, via asynchronous update, a class of multi-valued nearest neighbor maps F which better represent biological reality. States of F only change one at a time.

To make the collection of **RN**-compatible functions F smaller and more biologically realistic, we employ a switching system, which is an ODE system with discrete-valued interaction terms. They were introduced in the 1970's (Glass and Kauffman, 1972, 1973) as a continuous time counterpart to Boolean networks. A switching system is parameterized by continuous parameters, but this set decomposes into a finite number of computable regions (Cummins et al., 2016), each of which is associated with a single multi-level map *D*^*u*^ and its asynchronous update $F^{u}$, where $F^{u}$ is compatible with the switching system ODE (Crawford-Kahrl et al., 2018). The mutual position of these regions in the parameter space provide a natural way to define a notion of “neighboring” functions *D*^*u*^, *D*^*v*^ (and thus $F^{u},F^{v}$).

Our computational tool DSGRN (Cummins et al., 2016; Cummins et al., 2017; Harker, 2018) constructs the collection of all such parameter regions and encodes them in the form of a parameter graph. For each node *u* of the parameter graph, the DSGRN Database stores information about the global dynamics in form of a Morse graph, which is a summary of the dynamics of $F^{u}$. A DSGRN Database provides a summary of dynamics for all maps $F^{u}$ which are compatible with a switching system on **RN.** In this sense DSGRN represents the dynamics compatible with the network **RN** across all parameters.

DSGRN can be used to either list dynamical behaviors that are compatible with a given network **RN**, or search in the space of networks for those networks that provide most robustly dynamics of interest, for instance FC or FP.

## Author contributions

TG, KM conceptualized the paper. TG, BC wrote the paper. SH, BC implemented the methods and performed computations.

### Conflict of interest statement

The authors declare that the research was conducted in the absence of any commercial or financial relationships that could be construed as a potential conflict of interest.
